# Viral Transmission? A Longitudinal Study of Media Use and Its Relation to Mental Strain During the First 2 Years of the COVID-19 Pandemic

**DOI:** 10.1007/s12529-024-10293-3

**Published:** 2024-05-20

**Authors:** Antonia Bendau, Moritz Bruno Petzold, Andreas Ströhle, Jens Plag

**Affiliations:** 1 Department of Psychiatry and Neurosciences, CCM, Charité – Universitätsmedizin Berlin, corporate member of Freie Universität Berlin and Humboldt Universität zu Berlin, Charitéplatz 1, 10117 Berlin, Germany; 2 Department of Psychology, Institute for Mental Health and Behavioral Medicine, HMU Health and Medical University Potsdam, Potsdam, Germany; 3 Department of Psychology, MSB Medical School Berlin, Berlin, Germany; 4 Faculty of Medicine, HMU Health and Medical University Potsdam, Potsdam, Germany

**Keywords:** Doomsurfing, Doomscrolling, News, Anxiety, Fear

## Abstract

**Background:**

In light of the dynamic COVID-19 pandemic, the exposure to pandemic-related media coverage may change over time and may be particularly relevant due to associations with psychopathological symptoms. The aims of the present study were to examine changes in media consumption over time and to analyze its prospective associations with psychological strain.

**Method:**

The study uses a longitudinal observational design with ten periods of online data collection from March 2020 to April 2022 in an adult convenience sample (*N* = 8337) of the general population in Germany.

**Results:**

Our data revealed that the frequency and duration of pandemic-related media exposure as well as their subjective critical evaluation showed the highest levels at the beginning of the pandemic and peaked again in autumn 2020 and spring 2021. The primarily used media formats changed only slightly over time. The amount of media exposure at baseline was associated with more impairing pandemic-related anxiety 1 month, 1 year, and 2 years later.

**Conclusion:**

Our results hint to potentially problematical and long-lasting associations of pandemic-related media consumption with mental strain. Our findings could serve as an orientation for recommendations, further research, and adequate interventions for a responsible dealing with media coverage.

**Trial Registration:**

The authors have pre-registered this research at clinicaltrials.gov without an analysis plan; retrievable at: https://clinicaltrials.gov/ct2/show/NCT04331106.

**Supplementary Information:**

The online version contains supplementary material available at 10.1007/s12529-024-10293-3.

## Introduction

In the context of the COVID-19 pandemic, a congregation of two circumstances emerged that have never been encountered on this scale before: globally networked communities on the one side and a worldwide health crisis with a very significant socioeconomic impact on the other [1–6]. Thus, in light of the pandemic, the role of the media has become increasingly important not only as a central source of information but also as an essential platform for communication (e.g., with the potential to ameliorate interpersonal restrictions resulting from “social distancing”) [7–10]. This seems to be particularly relevant with regard to social media: in a Finish sample, for example, involvement in social media identity bubbles was associated with lower loneliness and less psychological distress — thus, it might offer meaningful social resources during times of physical distancing [8]. Additionally, recreational media use (e.g., binge watching series) may provide a source of joy and ameliorates boredom [11]. On the contrasting side, already in the early phase of the pandemic, population-based studies revealed that exposure to media sources disseminating information related to COVID-19 — including traditional print and online outlets, such as public and local TV, radio, and national and local newspapers [1, 12–15], as well as websites of official authorities [12, 14, 15], and social media platforms [1, 12, 14–16] — may also contribute to significant psychological strain. In these mainly cross-sectional trials, the self-reported frequency as well as the duration of topic-specific media usage (e.g., media coverage providing information about the virus and pandemic-related events) was found to be positively associated with the severity of stress-related psychopathological symptoms in adolescents [15] and adults [1, 12–14, 16].

*Mental strain*, or *psychological strain*, as examined in these studies, lacks a sharp definition but can be loosely described as a state of psychological distress arising from challenging situations that surpass an individual’s perceived coping capacities [1, 2, 12–17]. This strain manifests in various forms, including *emotional symptoms* like anxiety, irritability, sadness, and helplessness; *cognitive symptoms* such as persistent worry, weakened concentration, and impaired decision-making; and *physical symptoms* like fatigue and sleep disturbances. Pandemic-related studies focus predominantly on anxiety symptoms — e.g., characterized by worry, tension, and restlessness — and depressive symptoms, including low mood, anhedonia, lack of motivation, and lethargy. Alongside studies focusing on mental strain related to consuming media with dedicated pandemic-specific content, there is also research exploring the effects of media consumption during the pandemic, irrespective of its relation to pandemic-related information. For example, in a systematic review involving 13 studies and 760,474 adolescents [15] as well as in studies with US-American adults [16] and adults from the UK [1], more time spent engaging with social media, encompassing both pandemic-related and non-pandemic content, has consistently been linked to elevated levels of anxiety and depressive symptoms. In German and Italian samples, the use of social media as a source to obtain information about the pandemic was associated with higher perceived mental burden — mediated by increased stress symptoms [18]. In a Taiwanese study, the perceived risk of and worrying about COVID-19 constituted a mediator of the link between more pronounced search for COVID-19-related information on social media and internet news and lower subjective well-being [19]. In a three-wave panel study with 3912 adult Italians during the first months of the pandemic (April to July 2020), individuals with problematic social media use (irrespective of COVID-19-related content) reported, on average, heightened levels of psychological distress across the three assessment waves [20]. Aside from these effects on interpersonal level, no cross-lagged associations between changes in problematic social media use and subsequent changes in distress (and vice versa) were evident on within-person level [20]. This suggests that associations between social media use and psychological distress may be mainly driven by trait-like differences in media use and not by state-like individual changes over time [20]. However, the observation period of only 3 months might be too short to identify intraindividual changes over time and emphasizes the need for longer study intervals.

Whereas existing research mainly focuses on social media use [15, 21], it might be relevant to shed more light on other media formats, too — e.g., the use of official websites of government and health authorities. In cross-sectional data from the early pandemic, for example, official websites were the most frequently reported sources of information and showed more adaptive associations with mental strain than social media use [12]. However, neither potential changes in the use of different formats of media nor their associations with mental strain over the course of the pandemic were examined in detail so far.

Although the various primarily cross-sectional studies with data collections at the beginning of the pandemic are eligible to point out possible risk constellations and, therefore, provide an important base for timely recommendations of health authorities and quick decisions with respect to the individual handling of media usage, they are not able to consider the dynamic progress of the COVID-19 pandemic. For these reasons, periodically repeated measurements ideally within the same sample are needed in order to elucidate the different aspects of pandemic-related media consumption and its associations with mental strain. In this article, we therefore present longitudinal data from ten assessment periods over the first 2 years of the COVID-19 pandemic. This time frame encompasses several major waves of high infection rates in Germany and the first, second, and third officially ordered “lockdown” as well as periods of decreasing infection rates and eases of preventive measures in-between.

We aimed to complement and expand the findings of cross-sectional studies by relevant longitudinal insights through several research questions: (1) Are there descriptive changes over time with respect to the type, duration, and frequency of media usage and the perceived need to reduce it due to negative effects on mental health? (2) Does the duration and frequency of media consumption at baseline predict media consumption at later assessment waves? (3) Does using social media, respectively official websites of authorities, as a primary source of pandemic-related information at baseline predict its later perceived negative effects on mental health? (4) Does the duration and frequency of media consumption at baseline predict mental strain (i.e., COVID-19-related anxiety, limitations in daily life caused by pandemic-related anxiety, unspecific anxiety symptoms, and depressive symptoms; variables are selected based on previous studies [12]) at later assessment waves? (5) Do the levels of average media exposure that mark the difference between mild vs. moderate pandemic-related anxiety symptoms change over time?

## Methods

### Study Design

**Fig. 1:**
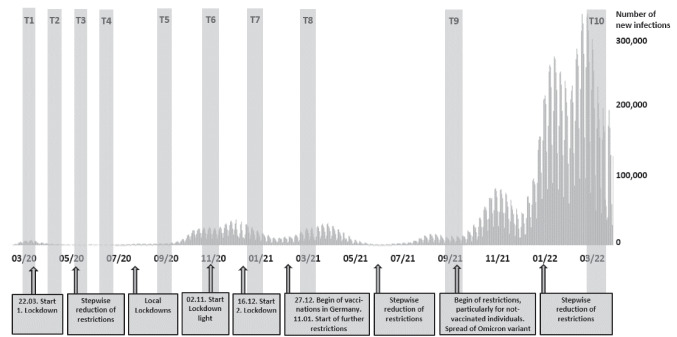
Overview of the 10 assessment waves (T1–T10), the daily rate of new infection, and relevant pandemic-related events and measures over time [2, 6, 20]. *Note.* T1, 27.03–06.04.2020, *n* = 5114; T2, 24.04–04.05.2020, *n* = 2567; T3, 15.05–25.05.2020, *n* = 1641; T4, 05.06–15.06.2020, *n* = 1411; T5, 25.09–05.10.2020, *n* = 1405; T6, 23.10–02.11.2020, *n* = 2225; T7, 01.01–11.01.2021, *n* = 1752; T8, 26.03–05.04.2021,* n* = 1578; T9, 24.09–04.10.2021, *n* = 1336; T10, 15.03–04.04.2022, *n* = 1529

A longitudinal observational online study with ten periods of data collection over the first 2 years of the COVID-19 pandemic (March/April 2020–March/April 2022) provided the data to examine changes in pandemic-related media exposure and its associations with anxiety and depressive symptoms [2, 22, 23]. A total of *N* = 8337 convenience-sampled adults from the general population in Germany participated in up to ten survey waves conducted via the SoSci-Survey platform. The number of participants varies between waves as individuals were able to choose which and how many measurements they wished to complete and consecutive participation in subsequent measurement points was not a prerequisite (see Fig. 1 for an overview of the ten survey waves and the pandemic situation). Non-probabilistic recruitment for the first participation was performed via news portals, social media channels (Twitter, Facebook, Instagram), and the homepage of the Charité—Universitätsmedizin Berlin [2, 22]. Participants, who gave their consent at first participation, were contacted for the next follow-up survey periods via e-mail (*n* = 3595). In addition, further first-time participants were recruited at T2 (3636 clicks on the survey link, 735 completed interviews), T3 (721 clicks, 216 completed interviews), T4 (130 clicks, 57 completed interviews), T5 (32 clicks, 12 completed interviews), and T6 (74 clicks, 32 completed interviews). Required for inclusion was a minimum age of 18 years, current residence in Germany, and the ability to complete the survey in German language. Prior to recruitment, the study was approved by the ethics committee of Charité - Universitätsmedizin Berlin (EA1/071/20) and registered on clinicaltrials.gov (NCT04331106). Data assessment was fully anonymous; data were stored separately from e-mail addresses and merged longitudinally via anonymous codes. Prior to participation, all participants gave informed consent. No financial or material incentives were issued for participation.

### Assessment

In all ten assessments, the basically same self-report questionnaire was used which required approximately 10 to 15 min to be completed [2, 22–24].

#### Media Consumption

The daily average of hours and the frequency (times per day) of media usage to obtain information about the COVID-19 topic were recorded at all assessments (“How much time (in hours) per day do you inform yourself about COVID-19 in the media?”; “How often per day do you inform yourself about COVID-19 in the media?”). Additionally, the survey included two items regarding the self-reflection of the subjective impact of media exploitation: “I think I should reduce media consumption related to the COVID-19 pandemic because it is very stressful for me” and “I already reduced media consumption related to the COVID-19 pandemic because it was very stressful for me” [12]. Both items were rated on a 5-point Likert scale, from 1 (“not true at all”) to 5 (“totally true”). Participants selected their primary source of pandemic-related information from a list of six available options (official government and health authority websites, news portals, social media, television, radio, and newspapers), with the possibility of selecting multiple choices.

#### Specific Pandemic-Related Anxiety

Furthermore, specific anxiety symptoms related to the pandemic were obtained with the COVID-19-Anxiety Questionnaire (C-19-A) [25], a scale adapted from a questionnaire for specific phobia (DSM-5 Severity Measure for Specific Phobia – Adult Scale). This modified scale comprises ten items that assess anxiety symptoms related to COVID-19, replacing the original questionnaire’s self-specified phobic stimuli or situations (e.g., “During the past 7 days, I have felt anxious, worried, or nervous about COVID-19” instead of “… about these situations”; for a detailed overview of all modifications and psychometric testing, see [25]). Each item is rated on a 5-point Likert scale from 0 (“never”) to 4 (“all the time”). The sum score categorizes pandemic-related phobic symptoms in none (0–4), mild (5–14), moderate (15–24), severe (25–34), and extreme (35–40) [25]. To capture the repercussions of COVID-19-related anxiety, we integrated a self-created item that assessed the extent to which this anxiety impaired participants’ daily life (“My anxiety concerning COVID-19 leads to limitations of my daily life”) on a 6-point Likert scale, from 1 (“not true at all”) to 6 (“totally true”). To maintain a succinct survey length, we did not specify which particular aspects of anxiety (such as fear of the immediate or long-term effects of infection, fear of restrictive measures) predominantly contributed to these limitations.

#### Unspecific Anxiety and Depressive Symptoms

The validated Patient Health Questionnaire-4 (PHQ-4) [26] was used to screen for symptoms of unspecific anxiety (two items: subscale GAD-2) and depression (two items: subscale PHQ-2). The item’s intensity was rated on a 4-point Likert scale from 0 (“not at all”) to 3 (“nearly every day”). The PHQ-4 sum score divides symptom severity in none (0–2), mild (3–5), moderate (6–8), and severe (9–12) symptoms [26].

### Analyses

All analyses were carried out with IBM SPSS Statistics version 29. The significance level was set to 0.05 (two-tailed) and missing data was handled by casewise deletion. Descriptive statistics were calculated for the frequency and duration of media use, the subjective need to reduce it, and the primarily used media sources for obtaining information about the pandemic. For inferential analyses, only three follow-up measurements were selected exemplary to keep the quantity of multiple tests low (T2, 1 month after baseline; T8, 1 year later; T10, 2 years later). Six separate linear regressions were conducted to analyze the longitudinal autocorrelations of (1) duration and (2) frequency of media use at baseline with their respective values (a) 1 month, (b) 1 year, and (c) 2 years later to examine the persistence of media use patterns over time, providing insights into whether initial media use habits predict similar behaviors in the future. Further, 24 separate longitudinal multiple linear regressions were performed using (1) duration and (2) frequency of media use at baseline as predictors, and mental strain — namely, (I) COVID-19-related anxiety, (II) limitations in daily life caused by pandemic-related anxiety, (III) unspecific anxiety symptoms, and (IV) depressive symptoms — as outcomes at (a) 1 month, (b) 1 year, and (c) 2 years later to analyze prospective associations of media use with mental strain. For the baseline T1-values of each respective mental strain, variable was controlled in these regressions to adjust for initial individual differences, thereby providing a more accurate examination of the changes in mental strain over time potentially attributable to media use. Six separate analyses of variance were carried out to test differences between individuals that (1) used vs. not used social media, respectively, and (2) used vs. not used official websites as a primary source of pandemic-related information at baseline (as two independent between-subject factors in one ANOVA) regarding the subjective need to reduce media consumption in (I) the future and (II) the past at (a) 1 month, (b) 1 year, and (c) 2 years later (with statistical control of the baseline values of the need to reduce). The Bonferroni-Holm correction was applied to the *p*-values of all inferential analyses to counterbalance multiple testing.

In a cross-sectional analysis early in the pandemic [12], a threshold of 7 times per day respectively 2.5 h of media consumption was identified as the point of differentiation between mild and moderate symptoms of mental strain. As internal and external circumstances may change over the course of the pandemic, these thresholds may also shift. Therefore, we expanded the descriptive examination [12] to 10 time points by calculating the average levels of media use for individuals with mild vs. moderate pandemic-related anxiety at each assessment wave and descriptively examining the values in-between as rough threshold estimates.

## Results

### Sample Characteristics

Of the *N* = 8337 participants, 71.7% (*n* = 5980) identified as female, 27.6% (*n* = 2297) as male, and 0.7% (*n* = 60) as diverse (for detailed sociodemographic and pandemic-related information at each measurement point, see Supplement Table S1). The mean age at initial participation in the study was 37.54 years (SD = 12.04; range, 18–99). Regarding their highest educational level, 53.3% of the sample reported a university degree, 30.1% a higher education entrance qualification, and 16.6% a secondary school degree or lower. A percentage of 9.5% suffered from (chronic) physical diseases (e.g., cancer, cardiovascular diseases, metabolic diseases). The percentages of individuals who knew people infected with COVID-19 (26.8% at T1; 98.7% at T10), had already experienced an infection themselves (0.9% at T1; 28.4% at T10), and had received at least one COVID-19 vaccination (24.3% at T8; 96.3% at T10) increased continuously over the 10 measurement points.

### Changes in the Usage of Media Regarding the COVID-19 Pandemic

**Fig. 2:**
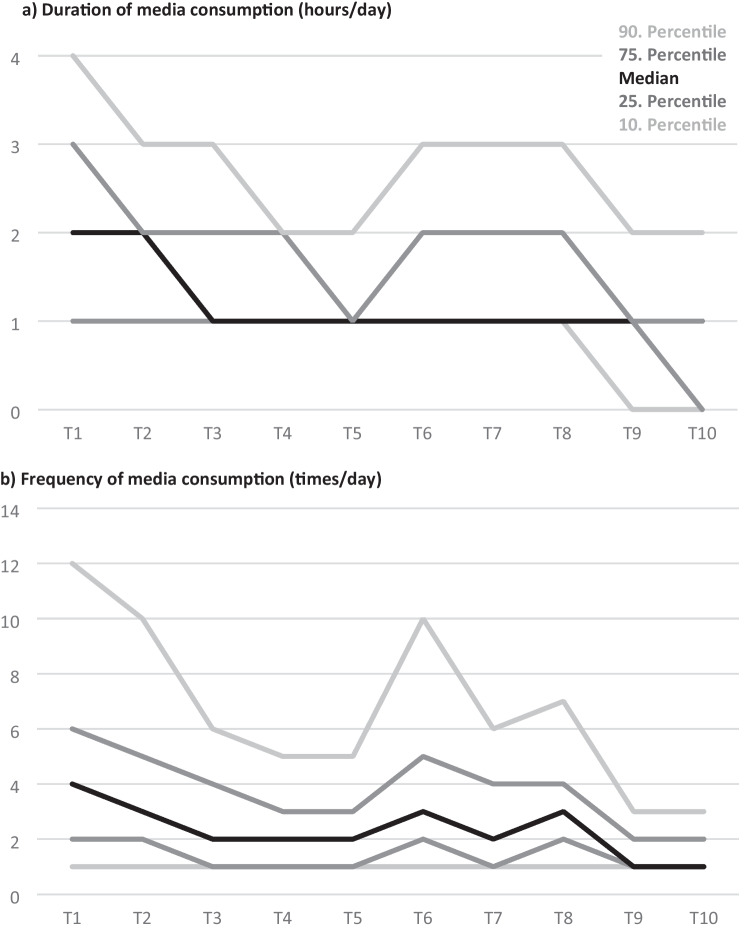
Course of **a** duration and **b** frequency of self-reported consumption of media regarding the COVID-19 pandemic over the first 2 years of the pandemic (T1–T10). *Note.* Sample sizes at the 10 assessment waves vary: T1, 5114; T2, 2548; T3, 1602; T4, 1382; T5, 1366; T6, 2145; T7, 1711; T8, 1545; T9, 1296; T10, 1482

Table 1 and Fig. 2 descriptively illustrate the changes in the self-reported daily duration and frequency of media consumption to obtain information about the pandemic (research question 1). The average duration as well as the average frequency peaked at the beginning of the pandemic in spring 2020, decreased continuously over the first four measurement periods, and increased again at the long-term follow-ups in autumn 2020 and spring 2021 (in parallel to steeply increasing case numbers of COVID-19 infections; see Fig. 1). The comparatively most pronounced changes were evident in those with particularly high levels of media consumption (see Fig. 2). These individuals particularly contributed to elevated mean values as can be approximatively deducted from the mostly lower median values.

**Table 1:** Media usage regarding the COVID-19 pandemic at the 10 assessment periods (T1–T10)

		**T1**	**T2**	**T3**	**T4**	**T5**	**T6**	**T7**	**T8**	**T9**	**T10**
	*N*	5114	2548	1602	1382	1366	2145	1711	1545	1296	1482
**Duration of media use (hours/day)**	***M***	**2.42**	**1.97**	**1.69**	**1.40**	**1.36**	**1.93**	**1.72**	**1.84**	**1.16**	**1.04**
	SD	2.05	1.53	1.49	1.14	1.58	1.73	1.69	1.71	1.30	1.56
	Median	2.00	2.00	1.00	1.00	1.00	1.00	1.00	1.00	1.00	1.00
**Frequency of media use (times/day)**	***M***	**7.32**	**4.62**	**4.16**	**2.59**	**3.00**	**4.65**	**3.68**	**3.98**	**1.86**	**1.78**
	SD	28.24	6.15	25.44	3.61	14.16	6.97	6.28	6.51	2.16	1.94
	Median	4.00	3.00	2.00	2.00	2.00	3.00	2.00	3.00	1.00	1.00
**Need to reduce (future)**	***M***	**2.73**	**2.56**	**2.24**	**1.99**	**1.96**	**2.53**	**2.43**	**2.62**	**1.91**	**1.93**
	SD	1.40	1.34	1.28	1.20	1.21	1.41	1.38	1.40	1.22	1.23
	Median	3.00	2.00	2.00	2.00	1.00	2.00	2.00	3.00	1.00	1.00
**Already reduced (past)**	***M***	**2.65**	**2.83**	**2.88**	**2.83**	**2.63**	**2.54**	**2.79**	**2.84**	**2.80**	**2.86**
	SD	1.49	1.49	1.51	1.54	1.52	1.46	1.49	1.49	1.53	1.56
	Median	2.00	3.00	3.00	3.00	2.00	2.00	3.00	3.00	3.00	3.00
**Sources of information (quantity)**	***M***	**3.09**	**2.91**	**2.93**	**2.90**	**2.95**	**3.19**	**3.12**	**3.15**	**2.88**	**2.81**
	SD	1.24	1.17	1.19	1.22	1.24	1.27	1.25	1.26	1.26	1.25
	Median	3.00	3.00	3.00	3.00	3.00	3.00	3.00	3.00	3.00	3.00

Scales of the need to reduce media consumption in the future as well as already reduced consumption in the past due to perceived negative effects on mental health range from 1 “not true at all” to 5 “absolutely true.” The quantity of primary used sources to obtain information about the pandemic ranges from 0 to 6 (official websites, news portals, social media, television, radio, newspaper)

**Fig. 3:**
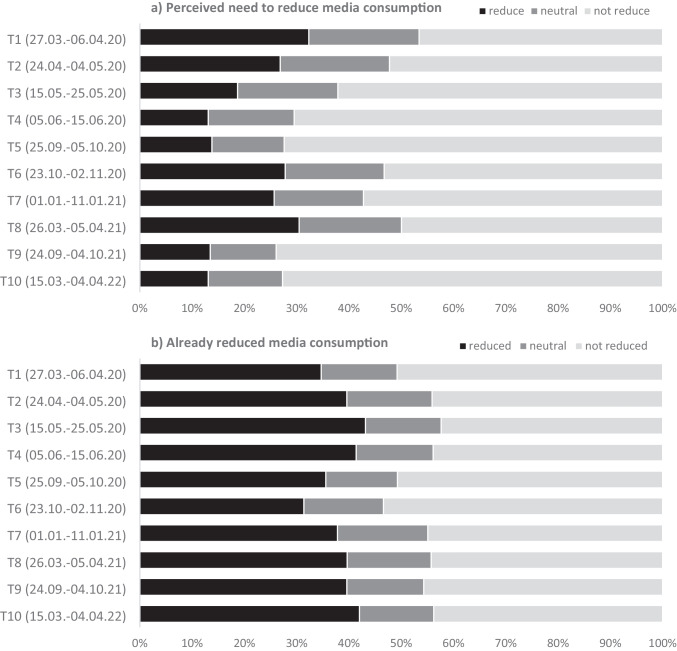
**a** Perceived need to reduce media consumption regarding the COVID-19 pandemic and **b** extent of already reduced media consumption because of its perceived adverse effects on mental health. *Note.* For simplified visualization, the original 5-point scale has been condensed to 3 categories, combining “slightly reduced” and “strongly reduced” into a single “reduced” category, and similarly, “not reduced” and “not reduced at all” into a single “not reduced” category. Sample sizes at the 10 assessment waves vary: T1, 5114; T2, 2548; T3, 1602; T4, 1382; T5, 1366; T6, 2145; T7, 1711; T8, 1545; T9, 1296; T10, 1482

The subjective need to reduce media exploitation because of its perceived negative effects on mental health (see Table 1 and Fig. 3) showed changes similar to the course of frequency and duration of media consumption. It exhibits its maximum at the early beginning of the pandemic and peaks again during autumn 2020 (second “wave” of COVID-19 infections and “lockdown” in Germany) and spring 2021 (third wave of infections and “lockdown” measures). The self-reported amount of already made reductions showed an inverse bimodal course: It peaked in summer 2020 and in spring 2022 — it might represent the time-lagged reaction on particularly high needs to prospectively reduce media consumption in the months/assessments before.

**Fig. 4:**
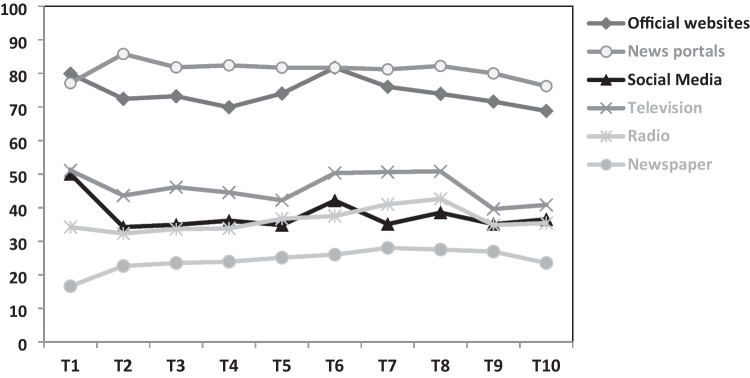
Percentages of self-reported primarily used sources to obtain information about the COVID-19 pandemic at the 10 assessment waves (T1–T10). *Note.* Percentages represent the relative proportion of individuals in the sample at each respective time point who indicated that they use the specified source (with the option for multiple selections available). Sample sizes at the 10 assessment waves vary: T1, 5114; T2, 2548; T3, 1602; T4, 1382; T5, 1366; T6, 2145; T7, 1711; T8, 1545; T9, 1296; T10, 1482

Figure 4 depicts which formats were used as primary information sources to obtain information regarding the pandemic. Official websites of the government and health authorities were the most commonly reported information source at T1 and peaked again in autumn 2020. At all follow-up assessments (T2–T10), the usage of news portals represented the most frequently reported source of information. The relative proportion of participants which used social media as a primary source to ascertain information about the pandemic decreased from baseline to the follow-ups, whereas the usage of radio and television was elevated from autumn 2020 to spring 2021. Newspapers were at all assessment periods reported the least but showed a growing trend over the course of the pandemic. In general, the reported percentages of different media formats exhibited only small changes — as well as the quantity of how many different formats were used (see Table 1).

Regarding the longitudinal autocorrelations of media use (research question 2), the duration (hours/day) of media consumption at baseline (T1) significantly predicted the duration 1 month (T2: *n* = 918; standardized *β* = .447, *p* < .001), 1 year (T8: *n* = 539; *β* = .433, *p* < .001), and 2 years later (T10: *n* = 460; *β* = .177, *p* < .001). Likewise, the frequency of media usage at baseline predicted the frequency 1 month (T2: *n* = 1335; standardized *β* = .196, *p* < .001), 1 year (T8: *n* = 861; *β* = .315, *p* < .001), and 2 years later (T10: *n* = 775; *β* = .262, *p* < .001).

Participants who reported to use social media as a primary information source at baseline showed, on average, a significantly stronger need to prospectively reduce their media consumption than individuals who not used social media for this purpose (controlling for baseline T1-values of the need to reduce) 1 month later (T2: *M* = 2.67, SD = 1.37 vs. *M* = 2.33, SD = 1.30; *F*(1, 1335) = 26.17, *p* < .001), 1 year later (T8: *M* = 2.77, SD = 1.43 vs. *M* = 2.62, SD = 1.40; *F*(1, 861) = 4.14, *p* = .042), and 2 years later (T10: *M* = 2.04, SD = 1.15 vs. *M* = 1.77, SD = 1.31; *F*(1, 775) = 12.16, *p* < .001; research question 3). No significant differences between users of official websites at T1 and individuals not using this information source were evident regarding the perceived need to reduce media usage in the future.

### Associations of Media Usage with Symptoms of Mental Strain

Supplement Table S1 provides the means and standard deviations of levels of mental strain at each assessment wave. The reliability of the scales was consistently good to excellent across all measurement points for COVID-19-related anxiety (Cronbach’s *α* of the C-19-A: T1, 0.86; T2, 0.87; T3, 0.89; T4, 0.88; T5, 0.88; T6, 0.88; T7, 0.88; T8, 0.90; T9, 0.89; T10, 0.89), unspecific anxiety (Cronbach’s *α* of the GAD-2: T1, 0.82; T2, 0.82; T3, 0.85; T4, 0.84; T5, 0.86; T6, 0.85; T7, 0.84; T8, 0.85; T9, 0.85; T10, 0.85), and depressive symptoms (Cronbach’s *α* of the PHQ-2: T1, 0.81; T2, 0.82; T3, 0.82; T4, 0.81; T5, 0.83; T6, 0.81; T7, 0.84; T8, 0.84; T9, 0.83; T10, 0.82).

Table 2 shows the results of multiple linear regressions of pandemic-related anxiety, unspecific anxiety, and depressive symptoms at the three follow-ups on frequency and duration of media consumption at baseline with partialization of the baseline values of symptom severity (research question 4). The average daily frequency of media use at baseline was significantly associated with higher pandemic-related anxiety 1 year later (T8). Further, higher frequency as well as longer duration of baseline media use was associated with significantly stronger perceived limitations in daily life because of pandemic-related anxiety 1 month (T2) and 1 year (T8) later; and, regarding the frequency as predictor, even 2 years later (T10). The broader constructs of unspecific anxiety and depressive symptoms at the follow-up measurements showed no significant associations with baseline media usage.

**Table 2:** Longitudinal associations of media usage at baseline (T1) with pandemic-related anxiety (C-19-A), perceived limitations in daily life due to this anxiety, and symptoms of unspecific anxiety (GAD-2) and depression (PHQ-4) at the follow-ups 1 month (T2), 1 year (T8), and 2 years (T10) later

	**Pandemic-related anxiety (C-19-A)**	**Anxiety leads to limitations in daily life**	**Unspecific anxiety symptoms (GAD-2)**	**Depressive symptoms (PHQ-2)**
**T1 media: Duration**				
T2 2020 (*n* = 1134)	*β* = .007 (*p* = .750)	*β* = .069 (*p* = .004*)	*β* = .013 (*p* = .553)	*β* = − .005 (*p* = .820)
T8 2021 (*n* = 736)	*β* = .064 (*p* = .035)	*β* = .096 (*p* = .003*)	*β* = − .029 (*p* = .327)	*β* = − .025 (*p* = .413)
T10 2022 (*n* = 657)	*β* = .045 (*p* = .192)	*β* = .078 (*p* = .030)	*β* = .003 (*p* = .935)	*β* = − .031 (*p* = .397)
**T1 media: Frequency**				
T2 2020 (*n* = 1341)	*β* = .045 (*p* = .014)	*β* = .063 (*p* = .004*)	*β* = .019 (*p* = .318)	*β* = − .019 (*p* = .335)
T8 2021 (*n* = 863)	***β*** = .109 (*p* < .001***)	***β*** = .102 (*p* < .001***)	*β* = .000 (*p* = .986)	*β* = − .002 (*p* = .949)
T10 2022 (*n* = 781)	*β* = .078 (*p* = .012)	***β*** = .144 (*p* < .001***)	*β* = .025 (*p* = .427)	*β* = .040 (*p* = .226)

Multiple linear regressions with control of baseline T1-values of the C-19-A, respectively perceived limitations, PHQ-2, and GAD-2 scores

Significance levels relate to Bonferroni-Holm corrected *p*-values; in the table, the uncorrected original *p*-values are displayed; *significant at .05 level, **significant at .01 level, ***significant at .001 level. T1, 27.03–06.04.2020; T2, 24.04–04.05.2020; T8, 26.03–05.04.2021; T10, 15.03–04.04.2022

Supplement Fig. S1 shows the average amount of media exposure for individuals with mild in comparison to those with moderate symptoms of pandemic-related anxiety during different stages of the pandemic (research question 5). The approximative descriptive thresholds were the highest at the beginning of the pandemic (2.5 h and 7 times/day) and peaked again from autumn 2020 to spring 2021. The comparatively lowest thresholds were evident at the last assessment (1.4 h and 2.5 times/day).

## Discussion

### Summary and Interpretation of the Results

The present study investigated the course of pandemic-related media exposure and its associations with pandemic-related anxiety, unspecific anxiety, and depressive symptoms among a large sample of adults from Germany during the first 2 years of the COVID-19 pandemic. In order to get a deeper insight into the predictive role of media usage for later mental strain (relative to baseline levels), we analyzed longitudinal associations of media consumption at baseline with later levels of mental strain. Frequency of baseline media use predicted higher pandemic-related anxiety 1 year later. Further, frequency and duration were both linked to significantly stronger perceived limitations in daily life because of pandemic-related fears 1 month and 1 year later (and, for frequency, even 2 years later). The broader constructs of unspecific anxiety and depressive symptoms at the follow-up measurements showed no significant associations with baseline media usage. Slight differences between the associations of pandemic-related fears with duration vs. frequency underline the importance to consider both indicators of media exposure. Since statements of the World Health Organization [3] and other public health authorities mainly recommend to reduce the frequency of media usage in order to maintain mental health, it seems to be necessary to appropriately incorporate the duration in recommendations as well.

At least for single variables and measurement points, our findings are congruent with preliminary data from cross-sectional trials suggesting a detrimental impact of media exposure on mental health. Additionally, also in line with previous cross-sectional data [12], our results suggest that media exploitation is stronger related to pandemic-specific anxiety than to broader unspecific constructs of stress-related mental symptoms. There exist different aspects that may constitute reasons for this: First, other factors than media exploitation (e.g., personal consequences of the pandemic or individual predisposition) might be more important for the development of unspecific anxiety and depressive symptoms. Second, from a statistical perspective, it is relevant that levels of unspecific anxiety and depression changed considerably less over time than specific pandemic-related fears [2, 24]. Thus, the explainable variance of the GAD-2 and PHQ-2 beyond its baseline values is far smaller than the variance of pandemic-related fears (and its perception as impairing daily life) which makes it unlikely to obtain statistically significant results regarding the broader constructs of general anxiety and depressive symptoms.

The average amount of the frequency and duration of media consumption to obtain information about the pandemic was the highest at the early beginning of the pandemic and peaked again at the second and third waves of high infection rates and strict “lockdown” measures in autumn 2020 and spring 2021. During the first assessment, the uncertainty and lack of information about the virus were probably extraordinarily high, and the strict lockdown measures may further enhance the need for both gaining information and keeping social communication with others [12]. In the subsequently following weeks and months, the levels of media usage decreased, probably explained by a reduced need for information because of a saturation of knowledge about the topic and eases of the pandemic situation (e.g., decreases of the number of infections and restrictive measures) [6]. The subsequent increases in media consumption from autumn 2020 on might be due to the reaggravation of the pandemic situation and new restrictive measures, which probably (a) enhanced the need for information about the virus and the restrictive rules and (b) refuelled the necessity of (social) media use to counterbalance physical distancing obligations and other limitations in daily life. Nevertheless, it did not reach the maximum amount of media usage at the start of the pandemic — maybe due to an already existing core level of basic knowledge that was already built during the initial phase of the pandemic. Remarkably, at all 8 assessments during the first year of the pandemic, the average values of media consumption prevailed above the official recommendations of the World Health Organization (e.g., a maximum of checking the media once or twice a day) [27]. The comparatively lowest levels of media consumption regarding the pandemic were evident at the last assessment — 2 years after the start of the pandemic. Besides familiarity with the pandemic situation and only few restrictive measures, this might be explained by other topics replacing the previously dominant medial presence of the pandemic in the media — e.g., the war in Ukraine [28]. Decreases from baseline to the first follow-ups were evident regarding the percentages of social media, television, and official websites serving as main sources of pandemic-related information. The less frequent usage of official websites may be explained by satiety of the active need and search for information. Alongside the rapid increase of infection rate and the second lockdown at the sixth assessment, this need seemed to rise again. The usage of news portals represented the most common source at all follow-ups. This might reflect major parts of the population progressively obtaining news about the pandemic rather unintentionally in the context of their general consumption of daily/weekly news. The same possible explanation may also apply for the slight increases in radio, newspapers, and television as primary sources of information across the pandemic. With regard to the decrease in the usage of social media from the first to second assessment, it is conceivable that this reduction was on average rather intentionally due to a rising awareness about the harmful effects of social media — particularly with respect to overflooding and misinformation [5, 21]. This assumption is underlined by former cross-sectional findings that the usage of social media as a primary source of information was associated with higher levels of COVID-19 specific and unspecific anxiety and depressive symptoms [12, 13]. Furthermore, our longitudinal data strengthens this hypothesis because the consumption of social media at baseline showed significant associations with a higher need to reduce media usage in the future (1 month, 1 year, and 2 years after baseline). Nevertheless, in summary, the percentages of different formats of media remained rather stable across the 2 years of the pandemic and exhibited only small changes. This may indicate that the primarily used sources of information reflect personal preferences and not so much situational circumstances. Thus, appropriate information and adaptive risk communication in the context of critical events should be optimally provided in all different media formats. Further, contrary to the mass media’s focus on negative events, it might be helpful to present also positive information — the according demand was expressed several times in a qualitative survey during the pandemic [22].

Congruent to cross-sectional results of our prior study [12], which showed that more frequent and longer media usage was associated with a higher subjective need to reduce media consumption, the reported levels of this need followed the same pattern as the frequency and duration in our longitudinal data. It was, on average, the highest at the early beginning of the pandemic, decreased subsequently, and peaked again at the second and third waves of infections, whereas it was the lowest at the last two assessments 1.5 years, respectively 2 years after the pandemic’s start. Already made reduction showed the inverse pattern. This seems reasonable because the perceived need to reduce probably triggers subsequent reductions (time-delayed evident in subsequent follow-ups) and, in turn, the need for further reductions may decrease the more reductions already have been made. It is noteworthy that at the 10 assessment points, the majority of individuals did not experience a pronounced need to reduce media consumption. This suggests either an absence of an aversive impact of media use on mental strain in these individuals or that such an impact was not subjectively perceived. Focusing on the subset of individuals who experience a maladaptive link between media use and mental strain may be particularly important.

We aimed to examine potential changes in approximative thresholds of media exploitation. The descriptive threshold separating mild from moderate symptom severity decreased across the first 3 months of the pandemic. A possible explanation may be that the vulnerability for burden-generating media consumption increases over time as a result of cumulative media-induced stress and leads to a reduced ability to compensate worrying news about the pandemic. Furthermore, it is conceivable that at the first weeks of the pandemic media was primarily used for obtaining important information about the virus and official measures in the context of the first “lockdown.” In contrast, during the ongoing pandemic, the need for basic knowledge may become gradually more saturated, and therefore, media consumption probably was driven by getting daily headlines from the news. This coverage might rather focus on dramatic developments and transport more emotionally loaded than pure and objective information. Therefore, a comparatively smaller amount of media usage may already be sufficient to produce malicious effects. In the later progress of the pandemic, the thresholds were temporarily higher again. In light of the parallel aggravation of the pandemic situation, the need for basic information may grow again and, moreover, the time in-between may have reduced some of the previously accumulated distress. In summary, our data indicates that — in contrast to existing literature — the situational context should be taken into account when critical thresholds are discussed with respect to a responsible usage of media.

With regard to potentially adverse effects of social media, besides scaremongering and emotionally loaded content, misinformation seems to constitute a major problem [5, 21]. A systematic review over 14 studies pointed out that misinformation circulating on social media was particularly strongly associated with psychological distress [21] and the proportion of COVID-19 misinformation on social media reached in a review of 22 studies up to 28.8% of posts [5]. Therefore, it is desirable to improve technical filters regarding misinformation and to train the population’s skills in identifying false or misleading information [5]. Further, some medial algorithms should be critically reflected because they may potentiate the effects of media exploitation by creating exuberant and biased bubbles of information on distinct topics [29].

In general, it seems to be important to target several levels to enhance an adaptive dealing with media. Besides aforementioned measures regarding media coverage itself, it is probably crucial to enable individuals to reflect and control their media usage and its consequences for mental health. Official recommendations can lay a basis for this and different professions and areas of daily life should try to foster this. For example, in a randomized-controlled trial, the experimental group which was instructed to reduce their daily social media use over a period of 2 weeks experienced significant decreases in depressive symptoms and pandemic-related burden, whereas life satisfaction and subjective happiness significantly increased (compared to the control group that did not receive any instructions) [14]. In addition, regular physical activity succeeded in ameliorating problematic social media use [30]. Individuals with deficient personal networks [16] or pre-existing vulnerability to fears and anxiety [12, 17] might be particularly considered since both were associated with higher and more burden-aggravating media use in previous studies.

Further, when reflecting media use, it might be relevant to differentiate between distinct motives to use (social) media: For example, a Belgian study showed that more anxious adolescents reported to use social media primarily to seek for strategies to adapt to the pandemic situation whereas more lonely individuals indicated coping with lacking social contact as main reason for the use of social media [7]. In a panel study with 1777 Czech adults [31] as well as in a US-American study [16], news consumption on social media platforms was the strongest predictor of exacerbated psychopathological symptoms. In contrast, the use of infotainment, together with an in-depth and contextual style of reading pandemic-related articles, was associated with better mental health [31].

### Strengths and Limitations

The key strength of our study is that we prospectively followed up a large sample of the general population in 10 assessment periods during the first 2 years of the COVID-19 pandemic in Germany. The early start of recruitment with baseline data when case numbers steeply increased and the monitoring during later stages of the pandemic allows reflecting crucial changes along the pandemic. The big sample size and the combination of different repeatedly assessed measurements of media usage and mental strain increase the value of our results.

However, our study is not without limitations. First, the sample was collected through convenience sampling methods, primarily via social media, which may not accurately represent the general German population. Across all survey waves, our sample was characterized by a higher proportion of female participants, a younger age distribution, and a higher education level compared to the general population. Furthermore, there might be a sampling bias with respect to a higher probability of participation for individuals that were more familiar with social media or had a greater interest in the topic. These factors could limit the generalizability of our results. Second, for reasons of feasibility, we used rather short self-report questionnaires. Those might be more vulnerable for answer biases and wrong subjective self-assessments and do not represent the constructs in their entirety. Third, no causal conclusions can be drawn from our longitudinal correlative-observational study design. Therefore, it is not clearly detectable to what extents media exposure affects psychological variables or whether elevated media usage is rather a result of mental distress and fulfills the role of a distress preserving and aggravating factor. Further, it cannot be ruled out that confounding variables may distort correlations or create spurious associations. Fourth, it is not entirely possible to differentiate between different formats of media sources because sometimes they are mixed up — e.g., when information of official authorities is implemented in social media channels.

## Conclusions

In summary, our findings provide some evidence for a persistent potentially maladaptive role of media exposure in the context of the COVID-19 pandemic from a longitudinal perspective. The frequency and the duration of media exploitation — particularly if exceeding a critical amount — might play an important role in the development and persistence of mental strain. Furthermore, our data substantiates the assumption that seeking for information and social exchange via media is for most individuals a rather normal reaction to an exceptional situation and decreases in most cases in parallel to a further relaxation of the situation and a growing familiarity with new circumstances. Nevertheless, it is important to critically observe and avoid exceeding critical thresholds since the amount of media consumption was, at least for some variables and assessment waves, found to be associated with higher levels of mental strain in a short- and long-term perspective. The pandemic’s highly dynamic situation requires dynamic information about the critical load of media exposure and its prospective consequences. Therefore, while acknowledging methodological limitations and considering the inconsistencies in predictive significance across different aspects of mental strain, our results may serve as a rough orientation for the general population, future studies, and recommendations by (mental) health professionals and official authorities. Furthermore, practical interventions to promote a responsible dealing with media coverage should be derived from the findings and be tested in adequate designs.

## Supplementary Information

Below is the link to the electronic supplementary material.

Supplementary file1 (DOCX 71 KB)

## Data Availability

The data are available from the corresponding author, upon reasonable request.

## Declarations

### Research Transparency Statement

The authors are willing to share their data, analytics methods, and study materials with other researchers. The material will be available upon reasonable request.

### Design and Analysis Transparency

The STROBE checklist for observational studies served as reporting guideline for our study.

## References

[1] Neill RD, Blair C, Best P, McGlinchey E, Armour C. Media consumption and mental health during COVID-19 lockdown: a UK cross-sectional study across England, Wales, Scotland and Northern Ireland. Z Gesundh Wiss. 2021;1–9. 10.1007/s10389-021-01506-0. 10.1007/s10389-021-01506-0 PMC7979466 33777650

[2] Bendau A, Asselmann E, Plag J, Petzold MB, Ströhle A. 1.5 years pandemic - psychological burden over the course of the COVID-19 pandemic in Germany: a nine-wave longitudinal community study. J Affect Disord. 2022;319:381–7. 10.1016/j.jad.2022.09.105. 10.1016/j.jad.2022.09.105 PMC9507788 36162668

[3] World Health Organization (WHO). Mental health and psychosocial considerations during the COVID-19 outbreak. 2020.

[4] Aggarwal K, Singh SK, Chopra M, Kumar S. Role of social media in the COVID-19 pandemic: a literature review. Data Mining Approaches for Big Data and Sentiment Analysis in Social Media. 2022;91–115.

[5] Gabarron E, Oyeyemi SO, Wynn R. COVID-19-related misinformation on social media: a systematic review. Bull World Health Organ. 2021;99:455-463A. 10.2471/BLT.20.276782. 34108756 10.2471/BLT.20.276782 PMC8164188

[6] World Health Organization (WHO). WHO health emergency dashboard. 2022.

[7] Cauberghe V, van Wesenbeeck I, de Jans S, Hudders L, Ponnet K. How adolescents use social media to cope with feelings of loneliness and anxiety during COVID-19 lockdown. Cyberpsychol Behav Soc Netw. 2021;24:250–7. 10.1089/cyber.2020.0478. 33185488 10.1089/cyber.2020.0478

[8] Latikka R, Koivula A, Oksa R, Savela N, Oksanen A. Loneliness and psychological distress before and during the COVID-19 pandemic: relationships with social media identity bubbles. Soc Sci Med. 2022;293:114674. 10.1016/j.socscimed.2021.114674. 34959045 10.1016/j.socscimed.2021.114674 PMC8688936

[9] Valdez D, ten Thij M, Bathina K, Rutter LA, Bollen J. Social media insights into US mental health during the COVID-19 pandemic: longitudinal analysis of Twitter data. J Med Internet Res. 2020;22:e21418. 33284783 10.2196/21418 PMC7744146

[10] Wu-Ouyang B, Hu Y. The effects of pandemic-related fear on social connectedness through social media use and self-disclosure. J Media Psychol. 2022. 10.1027/1864-1105/a000347.

[11] Erdmann E, Dienlin T. Binge-watching, self-determination, and well-being: a partially successful direct replication and extension of Granow et al. (2018). 2021.

[12] Bendau A, Petzold MB, Pyrkosch L, Mascarell Maricic L, Betzler F, Rogoll J, et al. Associations between COVID-19 related media consumption and symptoms of anxiety, depression and COVID-19 related fear in the general population in Germany. Eur Arch Psychiatry Clin Neurosci. 2021;271:283–91. 10.1007/s00406-020-01171-6. 32691135 10.1007/s00406-020-01171-6 PMC7371788

[13] Riehm KE, Holingue C, Kalb LG, Bennett D, Kapteyn A, Jiang Q, et al. Associations between media exposure and mental distress among US adults at the beginning of the COVID-19 pandemic. Am J Prev Med. 2020;59:630–8. 33011008 10.1016/j.amepre.2020.06.008 PMC7351429

[14] Brailovskaia J, Swarlik VJ, Grethe GA, Schillack H, Margraf J. Experimental longitudinal evidence for causal role of social media use and physical activity in COVID-19 burden and mental health. Z Gesundh Wiss. 2022;1–14. 10.1007/s10389-022-01751-x. 10.1007/s10389-022-01751-x PMC9437404 36068852

[15] Strasser MA, Sumner PJ, Meyer D. COVID-19 news consumption and distress in young people: a systematic review. J Affect Disord. 2022. 10.1016/j.jad.2022.01.007 PMC8742131 34990630

[16] Ren R, Yan B. Personal network protects, social media harms: evidence from two surveys during the COVID-19 pandemic. Front Psychol. 2022;13:964994. 10.3389/fpsyg.2022.964994. 36072053 10.3389/fpsyg.2022.964994 PMC9441876

[17] Bendau A, Kunas SL, Wyka S, Petzold MB, Plag J, Asselmann E, Ströhle A. Longitudinal changes of anxiety and depressive symptoms during the COVID-19 pandemic in Germany: the role of pre-existing anxiety, depressive, and other mental disorders. J Anxiety Disord. 2021;79:102377. 10.1016/j.janxdis.2021.102377. 33662702 10.1016/j.janxdis.2021.102377 PMC9758512

[18] Brailovskaia J, Cosci F, Mansueto G, Margraf J. The relationship between social media use, stress symptoms and burden caused by coronavirus (COVID-19) in Germany and Italy: a cross-sectional and longitudinal investigation. J Affect Dis Rep. 2021;3:100067. 10.1016/j.jadr.2020.100067 PMC8995101 35434690

[19] Luo Y-F, Shen H-Y, Yang S-C, Chen L-C. The relationships among anxiety, subjective well-being, media consumption, and safety-seeking behaviors during the COVID-19 epidemic. Int J Environ Res Public Health. 2021. 10.3390/ijerph182413189. 34948796 10.3390/ijerph182413189 PMC8700923

[20] Di Blasi M, Salerno L, Albano G, Caci B, Esposito G, Salcuni S, et al. A three-wave panel study on longitudinal relations between problematic social media use and psychological distress during the COVID-19 pandemic. Addict Behav. 2022;134:107430. 10.1016/j.addbeh.2022.107430. 35870439 10.1016/j.addbeh.2022.107430 PMC9287460

[21] Rocha YM, Moura GA de, Desidério GA, Oliveira CH de, Lourenço FD, Figueiredo Nicolete LD de. The impact of fake news on social media and its influence on health during the COVID-19 pandemic: a systematic review. Z Gesundh Wiss. 2021:1–10. 10.1007/s10389-021-01658-z. 10.1007/s10389-021-01658-z PMC8502082 34660175

[22] Bendau A, Plag J, Schulz L, Petzold MB, Ströhle A. Pandemic-associated consequences and need for support – a mixed-method longitudinal analysis over two years of the COVID-19-pandemic in Germany. Die Psychotherapie. 2023;2:106–15.

[23] Bendau A, Petzold MB, Plag J, Asselmann E, Ströhle A. Illness anxiety predicts higher mental strain and vaccine willingness-a nine‐wave longitudinal study during the first 1.5 years of the COVID‐19 pandemic in Germany. Stress Health. 2023. 10.1002/smi.3255 37158010

[24] Bendau A, Plag J, Kunas S, Wyka S, Ströhle A, Petzold MB. Longitudinal changes in anxiety and psychological distress, and associated risk and protective factors during the first three months of the COVID-19 pandemic in Germany. Brain Behav. 2021;11:e01964. 10.1002/brb3.1964. 33230969 10.1002/brb3.1964 PMC7744907

[25] Petzold MB, Bendau A, Plag J, Pyrkosch L, Maricic LM, Rogoll J, et al. Development of the COVID-19-Anxiety Questionnaire and first psychometric testing. BJPsych Open. 2020;6:e91. 10.1192/bjo.2020.82. 32812525 10.1192/bjo.2020.82 PMC7453355

[26] Löwe B, Wahl I, Rose M, Spitzer C, Glaesmer H, Wingenfeld K, et al. A 4-item measure of depression and anxiety: validation and standardization of the Patient Health Questionnaire-4 (PHQ-4) in the general population. J Affect Disord. 2010;122:86–95. 10.1016/j.jad.2009.06.019. 19616305 10.1016/j.jad.2009.06.019

[27] World Health Organization. Mental health considerations during COVID-19 outbreak. Geneva. 2020.

[28] Ramírez C, Durón RM. The Russia-Ukraine war could bring catastrophic public-health challenges beyond COVID-19. Int J Infect Dis. 2022. 10.1016/j.ijid.2022.04.016 PMC9004220 35427786

[29] Makhortykh M, Bastian M. Personalizing the war: perspectives for the adoption of news recommendation algorithms in the media coverage of the conflict in Eastern Ukraine. Media, War, Conflict. 2022;15:25–45. 10.1177/1750635220906254.

[30] Precht L-M, Stirnberg J, Margraf J, Brailovskaia J. Can physical activity foster mental health by preventing addictive social media use?–A longitudinal investigation during the COVID-19 pandemic in Germany. J Affect Dis Rep. 2022;8:100316 10.1016/j.jadr.2022.100316 PMC8824224 35165673

[31] Grygarová D, Adámek P, Juríčková V, Horáček J, Bakštein E, Fajnerová I, Kesner L. Impact of a long lockdown on mental health and the role of media use: web-based survey study. JMIR Mental Health. 2022;9:e36050. 35605112 10.2196/36050 PMC9277533

