# Supplementary material for: Viral Transmission? A Longitudinal Study of Media Use and Its Relation to Mental Strain During the First 2 Years of the COVID-19 Pandemic
Source: Int J Behav Med. 2024 May 20;32(2):214–26. doi: 10.1007/s12529-024-10293-3 (PMC12031861; doi:10.1007/s12529-024-10293-3)
Supplement: Supplementary file 1 — Supplementary file1 (DOCX 71 KB) [file 12529_2024_10293_MOESM1_ESM.docx]

**Supplement**

**Viral transmission? A longitudinal study of media use and its relation to**

**mental strain during the first two years of the COVID-19 pandemic**

**Table S1 – Demographics and mental strain of the subsamples at the ten assessment periods (T1 – T10)**

| **Point of measurement** | **T1  (*N* = 5114)** | **T2  (*N* = 2567)** | **T3  (*N* = 1641)** | **T4  (*N* = 1411)** | **T5  (*N* = 1405)** | **T6  (*N* = 2225)** | **T7  (*N* = 1752)** | **T8 (*N* = 1578)** | **T9  (*N* = 1336)** | **T10 (*N* = 1529)** |
| --- | --- | --- | --- | --- | --- | --- | --- | --- | --- | --- |
| **Characteristic** | **Mean (SD) Range** | **Mean (SD) Range** | **Mean (SD) Range** | **Mean (SD) Range** | **Mean (SD) Range** | **Mean (SD) Range** | **Mean (SD) Range** | **Mean (SD) Range** | **Mean (SD) Range** | **Mean (SD) Range** |
| **Age (years)** | 36.6 (11.5); 18 – 99 | 38.0 (12.2); 18 – 81 | 39.8 (12.3); 18 – 76 | 40.4 (12.5); 18 –77 | 40.6 (12.3)  18 – 77 | 40.4 (12.3)  18 – 82 | 41.2 (12.6); 18 – 84 | 41.5 (12.4); 18 – 84 | 40.7 (12.3)  18 – 77 | 42.1(12.4)  18 – 84 |
| **Characteristic** | ***N* (%)** | ***N* (%)** | ***N* (%)** | ***N* (%)** | ***N* (%)** | ***N* (%)** | ***N* (%)** | ***N* (%)** | ***N* (%)** | ***N* (%)** |
| **Gender**  • Female  • Male  • Diverse | 3618 (70.7) 1457 (28.5) 39 (0.8) | 1927 (75.1)  628 (24.5)  12 (0.5) | 1255 (76.5)  377 (23.0)  9 (0.5) | 1087 (77.0) 315 (22.3) 9 (0.6) | 1081 (76.9) 314 (22.3) 10 (0.7) | 1719 (77.3) 488 (21.9)  18 (0.8) | 1356(77.4)  383 (21.9)  13 (0.7) | 1222 (77.4) 344 (21.8) 12 (0.8) | 1048 (78.4) 279 (20.9) 9 (0.7) | 1177 (77.0) 343 (22.4)  9 (0.6) |
| **Relationship status**  • Single, widowed, divorced  • Solid relationship  • Married  • Other | 1867 (36.5) 1620 (31.7) 1542 (30.2) 85 (1.7) | 872 (34.0)  808 (31.5)  858 (33.4)  22 (0.9) | 587 (35.8)  471 (28.7)  576 (35.1)  7 (0.4) | 512 (36.3) 389 (27.6) 499 (35.4) 11 (0.8) | 499 (35.5) 392 (27.9) 502 (35.7) 12 (0.9) | 761 (34.2) 623 (28.0) 811 (36.4)  30 (1.3) | 576 (32.9)  482 (27.5)  676 (38.6)  18 (1.0) | 518 (32.8) 425 (26.9) 622 (39.4) 13 (0.8) | 442 (33.1) 365 (27.3) 518 (38.8) 11 (0.8) | 474 (31.0) 402 (26.3) 643 (42.1)  10 (0.8) |
| **Education level**  • University degree  • Higher educ. entrance  • Secondary degree  • Lower secondary degree  • No school degree | 2602 (50.9) 1631 (31.9) 768 (15.0) 98 (1.9) 15 (0.3) | 1541 (60.0)  718 (28.0)  214 (8.3)  23 (0.9)  4 (0.2) | 944 (57.5)  433 (26.4)  154 (9.4)  17 (1.0)  6 (0.4) | 820 (58.1) 374 (26.5) 137 (9.7) 14 (1.0) 3 (0.2) | 789 (56.2) 373 (26.5) 133 (9.5) 15 (1.1) 3 (0.2) | 1174 (52.8) 676 (30.4) 331 (14.9) 37 (1.7) 7 (0.3) | 1047 (59.8)  443 (25.3)  232 (13.2)  28 (1.6)  2 (0.1) | 936 (59.3) 403 (25.5) 209 (13.2) 27 (1.7) 3 (0.2) | 834 (62.4) 325 (24.3) 153 (11.5) 22 (1.6) 2 (0.1) | 902 (59.0) 394 (25.8) 210 (13.7) 19 (1.2) 4 (0.3) |
| **Physical disease**  • Yes  • No | 562 (11.0) 4552 (89.0) | 249 (9.7)  2220 (86.5) | 182 (11.1) 1321 (80.5) | 171 (12.4) 1130 (80.1) | 160 (11.4) 1055 (75.1) | 271 (12.2) 1742 (78.3) | 195 (11.1) 1280 (73.1) | 179 (11.4) 1132 (71.7) | 147 (11.0) 878 (65.7) | 170 (11.1) 998 (65.3) |
| **Confrontation with COVID-19** |  |  |  |  |  |  |  |  |  |  |
| **Knows an infected individual** |  |  |  |  |  |  |  |  |  |  |
| • Yes  • No | 1368(26.8)  3728 (73.2) | 944 (36.8)  1605 (62.5) | 659 (40.2)  973 (59.3) | 581 (41.2) 821 (58.2) | 729 (51.9) 669 (47.6) | 1330 (59.8) 887 (39.9) | 1450 (82.8)  295 (16.8) | 51357 (86.0) 212 (13.4) | 1166 (78.3) 164 (12.3) | 1504 (98.4) 20 (1.3) |
| **Was diagnosed with COVID-19** |  |  |  |  |  |  |  |  |  |  |
| • Yes  • No | 44 (0.9)  5034 (99.1) | 24 (0.9)  2521 (98.2) | 13 (0.8)  1619 (98.7) | 7 (0.5)  1389 (98.4) | 19 (1.4) 1378 (98.1) | 38 (1.7) 2215 (97.8) | 72 (4.1)  1673 (95.5) | 85 (5.4)  1481 (93.9) | 74 (5.5) 1254 (93.9) | 433 (28.3) 1092 (71.4) |
| **Currently in quarantine** |  |  |  |  |  |  |  |  |  |  |
| • Yes  • No | 355 (6.9)  4693(90.7) | 71 (2.8)  2441 (95.1) | 27 (1.6)  1587 (96.7) | 13 (0.9)  1370 (97.1) | 19 (1.4) 1359 (96.7) | 70 (3.1) 2108 (94.7) | 44 (2.5)  1682 (96.0) | 33 (2.1)  1506 (95.4) | 15 (1.1) 1297 (97.1) | 86 (5.6) 1413 (92.4) |
| **Characteristic** | **Mean (SD)** | **Mean (SD)** | **Mean (SD)** | **Mean (SD)** | **Mean (SD)** | **Mean (SD)** | **Mean (SD)** | **Mean (SD)** | **Mean (SD)** | **Mean (SD)** |
| **Mental strain*** |  |  |  |  |  |  |  |  |  |  |
| **COVID-19 anxiety (C-19-A)** | 10.1 (6.9) | 8.3 (6.4) | 7.1 (6.3) | 5.7 (5.6) | 5.8 (5.5) | 8.9 (6.8) | 8.5 (6.8) | 8.7 (7.2) | 4.1 (4.8) | 5.4 (5.8) |
| **Limitations in daily live** | 2.4 (1.5) | 2.2 (1.4) | 2.1 (1.4) | 2.0 (1.3) | 1.9 (1.3) | 2.3 (1.5) | 2.2 (1.4) | 2.3 (1.5) | 1.8 (1.2) | 1.9 (1.3) |
| **Unspecific anxiety (GAD-2)** | 2.0 (1.8) | 1.9 (1.7) | 1.7 (1.7) | 1.6 (1.6) | 1.6 (1.6) | 1.9 (1.7) | 1.7 (1.7) | 1.9 (1.8) | 1.5 (1.6) | 1.5 (1.6) |
| **Depressive Symptoms (PHQ-2)** | 2.1 (1.7) | 2.1 (1.6) | 1.9 (1.7) | 1.8 (1.7) | 1.7 (1.6) | 2.0 (1.7) | 2.0 (1.7) | 2.3 (1.7) | 1.6 (1.6) | 1.7 (1.6) |

*Note. Higher scores indicate higher levels of mental strain (C-19-A: scale ranges from 0 to 40; limitations: 1-6; GAD-2: 0-6; PHQ-2: 0-6)

**Figure S1.** Approximate descriptive threshold of the average amount of media consumption to ascertain information about the pandemic that marks the transition between mild and moderate symptoms of pandemic-related anxiety (C-19-A) across the ten assessment waves (T1 - T10).

**~ 5.0**

**~ 7.0**

**~ 1.4**
